# Phylogenetic analysis reveals dynamic evolution of the poly(A)-binding protein gene family in plants

**DOI:** 10.1186/s12862-014-0238-4

**Published:** 2014-11-25

**Authors:** Daniel R Gallie, Renyi Liu

**Affiliations:** Department of Biochemistry, University of California, Riverside, CA 92521-0129 USA; Department of Botany and Plant Sciences, University of California, Riverside, CA 92521-0129 USA

**Keywords:** PABP, eIF4G, eIF4B, Translation initiation, Protein synthesis, Gene duplication

## Abstract

**Background:**

The poly(A)-binding protein (PABP) binds the poly(A) tail of eukaryotic mRNAs and functions to maintain the integrity of the mRNA while promoting protein synthesis through its interaction with eukaryotic translation initiation factor (eIF) 4G and eIF4B. PABP is encoded by a single gene in yeast and marine algae but during plant evolution the PABP gene family expanded substantially, underwent sequence divergence into three subclasses, and acquired tissue-specificity in gene family member expression. Although such changes suggest functional specialization, the size of the family and its sequence divergence have complicated an understanding of which gene family members may be foundational and which may represent more recent expansions of the family to meet the specific needs of speciation. Here, we examine the evolution of the plant PABP gene family to provide insight into these aspects of the family that may yield clues into the function of individual family members.

**Results:**

The PABP gene family had expanded to two members by the appearance of fresh water algae and four members in non-vascular plants. In lycophytes, the first sequence divergence yielding a specific class member occurs. The earliest members of the gene family share greatest similarity to those modern members whose expression is confined to reproductive tissues, suggesting that supporting reproductive-associated gene expression is the most conserved function of this family. A family member sharing similarity to modern vegetative-associated members first appears in gymnosperms. Further elaboration of the reproductive-associated and vegetative-associated members occurred during the evolution of flowering plants.

**Conclusions:**

Expansion of the plant PABP gene family began prior to the colonization of land. By the evolution of lycophytes, the first class member whose expression is confined to reproductive tissues in higher plants had appeared. A second class member whose expression is vegetative-associated appeared in gymnosperms and all three modern classes had fully evolved by the appearance of the first known basal angiosperm. The size of each PABP class underwent further expansion during subsequent evolution, especially in the Brassicaceae, suggesting that the family is undergoing dynamic evolution.

**Electronic supplementary material:**

The online version of this article (doi:10.1186/s12862-014-0238-4) contains supplementary material, which is available to authorized users.

## Background

Most eukaryotic cellular mRNAs contain a 5′ cap structure and a poly(A) tail. The poly(A) tail is added to a nascent transcript during nuclear pre-mRNA processing. The average length of a poly (A) tail varies depending on the species, ranging from 75–90 adenosine residues in yeast to 200–300 residues in mammals [1]. Binding of the poly(A)-binding protein (PABP) to a poly(A) tail requires a minimum of 12 adenosine residues although one molecule of the protein occupies approximately 25 adenosine residues. PABP contains four tandem copies of an RNA recognition motif (RRM) that make direct contact with poly(A) RNA and a C-terminal region involved in self interaction and packing density [2-5]. The average poly(A) tail, therefore, can support binding of multiple PABP molecules [2,6].

The 5′-cap serves as the binding site for eIF4F, a complex consisting of eIF4E, the subunit that binds the 5′-cap; eIF4G, a modular scaffold protein involved in multiple protein interactions; and eIF4A, an ATP-dependent RNA helicase. eIF4G recruits additional factors including eIF4B, PABP, and eIF3. eIF4F, eIF4A, and eIF4B use the energy provided by ATP hydrolysis to unwind any secondary structure present in the 5′-leader to prevent it from inhibiting 40 S scanning during its search for the initiation codon [7]. In a step requiring an interaction between eIF4G and eIF3, the ribosomal 43 S complex is recruited to scan the 5′-leader in order to locate the initiation codon whereupon the 60 S subunit joins to form the translationally competent 80 S ribosome.

The 5′-cap and poly(A) tail stimulate translation synergistically which is mediated by a physical interaction between PABP bound to the poly(A) tail and the cap binding complex [8-11]. An interaction between PABP and eIF4G is conserved in plants, animals, and yeast while an interaction between PABP and eIF4B, a factor that increases the helicase activity of eIF4A, is conserved in plants and animals [9,10,12-20]. The RRM1 and RRM2 of mammalian and yeast PABP are required to interact with eIF4G [9,13,21,22] whereas the RRM1 of plant PABP is sufficient to interact with eIF4G [18]. Plant eIF4B also interacts within the RRM1 of PABP and its binding site overlaps with the binding site for eIFiso4G (a plant isoform of eIF4G) [18]. The interaction between PABP and eIF4G results in the pseudocircularization of an mRNA [11] which in turn stimulates translation by promoting recruitment of the 43 S complex to the mRNA [9]. Although subsequent electron microscopic studies of polysomes formed in translation lysate have supported mRNA pseudocircularization [23,24], recent analysis using electron tomography of polysomes in intact cells has suggested that densely packed, helical array of ribosomes in a large polysome may spatially constrain the interaction of the termini and inhibit circularization [25].

PABP is a highly conserved protein throughout eukaryotes and, in addition to its role in translation, it is involved in mRNA biogenesis, mRNA transport, and mRNA stability [26]. In yeast, PABP is required for poly(A) nuclease (PAN)-mediated deadenylation whereas it inhibits Caf1/Not1-mediated deadenylation [27,28]. PABP also inhibits deadenylation in mammals [29,30] and in *Xenopus* oocytes [31]. In yeast, where mRNA degradation occurs largely through a deadenylation-dependent decapping pathway [28,32], PABP also promotes mRNA stability by inhibiting DCP1/2-mediated decapping [27,28]. Loss of PABP following deadenylation can destabilize the association of eIF4F with the 5′-cap, rendering the cap more susceptible to attack by DCP1/2. Inhibition of decapping by PABP is also observed in mammals [33]. The exosome, which contains multiple subunits exhibiting exoribonuclease activity involved in RNA 3′ → 5′ degradation, is stimulated by PABP, suggesting that the exosome complex may interact with PABP [34].

While yeast expresses just a single PABP protein which is essential [2], PABP in plants and many animal species is expressed by a multigene family. For example, four full length PABP genes are present in the human genome [26] whereas eight are present in Arabidopsis [35]. The presence of a large and divergent gene family, in which members are co-expressed, raises the possibility that plant PABP isoforms have functionally specialized. Initial studies of a few members of the Arabidopsis PABP gene family have supported this possibility. Expression of Arabidopsis PAB2, PAB3, and PAB5 in a yeast *pab1* null mutant rescued the viability of the mutant [36-38]. PAB2 and PAB5 promote translation initiation and poly(A) shortening in yeast [36,37]. PAB2, but not PAB5, protected the mRNA 5′-cap against decapping in yeast [36,37] and PAB2 can interact with yeast eIF4G [37].

PAB3 fails to perform two of the known functions of yeast Pab1p in that it does not protect the mRNA 5′-cap from decapping in yeast and it does not interact with yeast eIF4G [38]. Instead, Arabidopsis PAB3 accelerated the entry of mRNA into the degradation pathway and the entry of mRNA into the translated pool in yeast. PABP shuttles between the nucleus and the cytoplasm in an Xpo1p-dependent manner in *Saccharomyces cerevisiae* [39] and in *Schizosaccharomyces pombe* [40], a fraction of yeast Pab1p localizes to the nucleus and associates with cleavage factor IA (CFIA) (*RNA15*) involved in 3′-end formation [41]. A fraction (7%) of Arabidopsis PAB3 also localizes to the nucleus when expressed in yeast and functions in mRNA biogenesis [42]. PAB3 partially restored PAN-dependent control of the length of the poly(A) tail during mRNA biogenesis in a yeast *pab1* null mutant. PAB3 is synthetic lethal with *rna15-2*, an allele of the gene encoding the Rna15p subunit of the nuclear factor CFIA, which is required for pre-mRNA cleavage, as well as polyadenylation [43]. PAB3 is also synthetic lethal with *gle2-1*, a mutant allele of the gene encoding the nuclear pore complex (NPC)-associated protein. Synthetic lethality between the nuclear function of PAB3 and Gle2p suggests the inner face of the NPC as a possible site of PAB3 action in the nucleus. Overexpression of PAB3 in yeast alleviated the mRNA export block of the *nab2-1* mutant strain, which is defective in one of the shuttling hnRNP proteins required for mRNA export. Thus, PAB3 is incapable of supporting poly(A)-dependent translation or the interaction between the 5′-cap and the poly(A) tail required for the synergistic stimulation of translation in yeast. However, PAB3 does correct the temporal lag prior to the entry of mRNA into the decay pathway in *pab1* mutant yeast, but only partially, suggesting only partial function in mRNA decay [38]. These findings suggest that PAB3 functions in the nucleus by facilitating mRNA biogenesis and export in yeast. The observation that PAB3 does not protect the mRNA 5′-cap in yeast and fails to interact with yeast eIF4G could indicate either that PAB3 does not participate in these processes or that it is unable to interact with the yeast factors in each pathway.

Despite these analyses of plant PABP functions in yeast, the underlying biological significance of the expansion and divergence of the PABP gene family in plants remains unknown. In this study, we have examined the evolution of plant PABP gene family from the single gene present in marine algae to the large and diverse family present in land plants. We observe that expansion of the PABP gene family occurred by the appearance of charophytes (fresh water algae) and these are most similar to those family members whose expression in confined to reproductive tissues, suggesting that supporting reproductive-associated gene expression is the most conserved function of the gene family. Vegetative-associated members of the family appeared with the evolution of seed plants with further expansion of the family during subsequent evolution of flowering plants.

## Results

### The PABP gene family in higher plants is composed of highly divergent member classes

**Figure 1:**
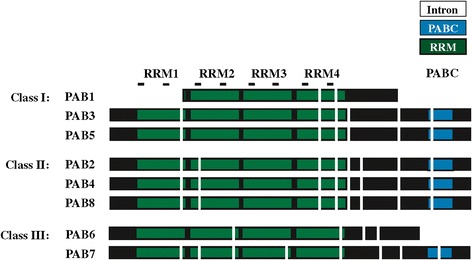
**Domain organization of the PABP gene family in** ***Arabidopsis thaliana*** **.** Each RNA recognition motif (RRM) is indicated in green and the PABC domain is indicated in blue. The position of each intron is indicated by vertical white bars. The RNP1 and RNP2 motifs within each RRM are indicated as lines above each domain. Protein sequences used were: PAB1 (At1g34140), PAB2 (At4g34110), PAB3 (At1g22760), PAB4 (At2g23350), PAB5 (At1g71770), PAB6 (At3g16380), PAB7 (At2g36660), and PAB8 (At1g49760).

The PABP gene family of *Arabidopsis thaliana*, a widely used model for flowering plants, is composed of eight genes. As the PABP domain structure is conserved throughout eukaryotes, these eight members contain tandemly-arranged RRMs characteristic of this protein (Figure 1). Each RRM is more highly conserved with the corresponding RRM of PABP proteins from other species than to the other RRMs within the same protein [44], indicating that domain duplication occurred prior to speciation. The members of the gene family largely differ, however, in the C-terminal region which has been implicated in self-association [3-5]. The C-terminal region contains a PABC domain required for interaction with partner proteins containing a PAM2 motif that interacts directly with the PABC domain [26].

**Figure 2:**
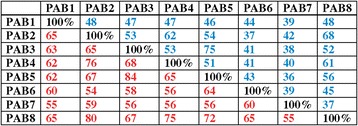
**Sequence identity and similarity of the PABP gene family members in** ***Arabidopsis thaliana*** **.** Comparison of the amino acid sequence conservation among the Arabidopsis PABP gene family members. Numbers in blue represent percent identity whereas numbers in red represent percent similarity.

Based on differences in sequence and expression specificity, the eight PABP genes were classified into four groups [35]. Expression of class I genes (PAB3 and PAB5) is restricted to reproductive tissues while class II genes (PAB2, PAB4, and PAB8) are widely expressed [35-37,45]. Class III genes (PAB6 and PAB7) exhibit weak and restricted expression whereas the class IV gene, PAB1, is expressed mostly in roots and was designated an “orphan gene” [35,46]. PAB1 lacks the RRM1 and PABC domain entirely while the PABC domain is missing in PAB6 (Figure 1). Most members of the family share less than 50% identity with the other family members (Figure 2). PAB7 and PAB6 share the least overall identity or similarity with other members of the family with PAB1 nearly as divergent. The class I members, i.e., PAB3 and PAB5, share the greatest similarity with each other and the class II members, i.e., PAB2, PAB4, and PAB8, share greater similarity with one another than with any other member (Figure 2).

### Expansion of the progenitor PABP gene family began prior to the evolution of land plants

**Figure 3:**
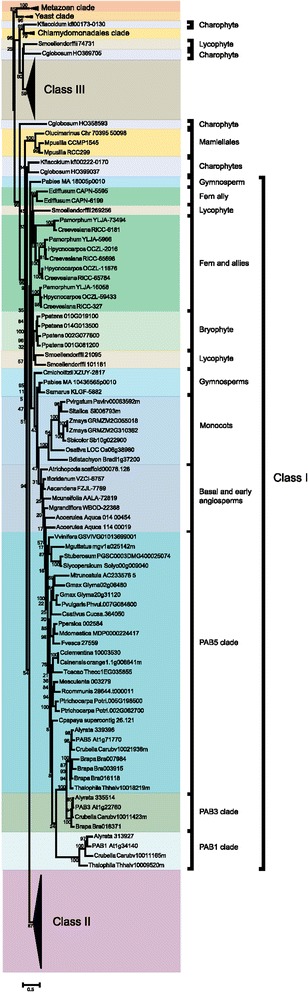
**Phylogenetic analysis of class I PABP proteins in plants.** A phylogenetic tree of class I PABP proteins in plants was constructed using the maximum-likelihood method. Numbers on each branch denote percentages of bootstrap support.

To determine how the family evolved from a single gene in yeast and algae to a large family in land plants composed of highly diverged subclasses, phylogenetic analysis was performed using available sequences from throughout plant evolution. The tree was rooted with the single gene sequences from *S. cerevisiae* and *S. pombe* and the four PABP sequences from the human genome. By the appearance of early land plants such as the bryophyte *Physcomitrella patens*, the PABP gene family had expanded to four, highly similar members that form two subgroups (Figure 3 and Additional file 1), suggesting that a single PABP progenitor gene had duplicated and each of these underwent an additional duplication. The four *P. patens* proteins cluster with the class I members of *A. thaliana*, suggesting that the class I proteins may represent the foundational members of the land plant PABP family.

**Figure 4:**
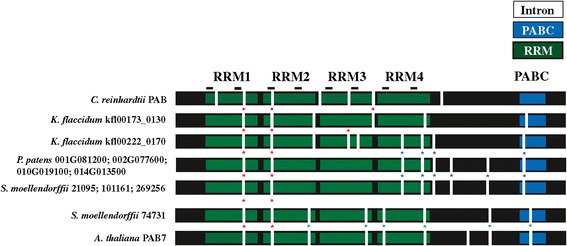
**A class III gene first appears in vascular plants.** RRMs (green), PABC domain (blue), RNP1 and RNP2 motifs (lines), and introns (vertical white bars) are indicated for the single PABP gene of *C. reinhardtii*, the two genes of *K. flaccidum*, the four progenitor PABP genes of *P. patens*, and the four genes of *S. moellendorffii. A. thaliana* PAB7 (At2g36660) is included for comparison. Ancestral introns are indicated with red asterisks. Introns unique to class I/II progenitor PABP genes or class III (PAB7-like) genes are indicated with blue or green asterisks, respectively.

As land plants evolved from aquatic ancestors that are sister groups to charophycean green algae (i.e., fresh water algae) (see Additional file 2), we examined the available PABP sequences in these species. The genome sequence of the charophyte *Klebsormidium flaccidum* representing the Klebsormidiophyceae was recently reported [47]. A search of the *K. flaccidum* genome revealed the presence of two genes encoding PABP and a search of charophyte ESTs identified partial but distinct sequences from *Chaetosphaeridium globosum*, which was the species in which the longest partial EST sequences were available. *K. flaccidum* kfl00222-0170 and the *C. globosum* HO399037 and HO358593 ESTs were more related to PABP proteins within the class I/II groups than they were to class III proteins whereas *K. flaccidum* kfl00173_0130 clustered with the marine algal PABPs (Figure 3 and Additional file 1). Three of the introns in *K. flaccidum* kfl00222_0170 and kfl00173_0130 are conserved with those of the salt-water algal species, *Chlamydomonas reinhardtii*, representing the Chlamydomonadales (Figure 4). Two of these three introns are conserved with PABP genes of higher plants and six of the nine introns in kfl00222_0170 are conserved with those in the four *P. patens* genes whereas only two of the six introns in kfl00173_0130 are conserved with the *P. patens* genes (Figure 4). The two introns present in the C-terminal region of the four *P. patens* genes are not present in kfl00222_0170 and therefore likely appear first in *P. patens* and are maintained during subsequent PABP gene evolution. However, the intron pattern of what would become class I and II genes had largely evolved by the appearance of *K. flaccidum* kfl00222_0170.

Although the phylogenetic analysis included the single PABP homolog present in the marine algal species of the Chlamydomonadales such as *C. reinhardtii* and *V. carteri* homologs with class III proteins while the single PABP homolog present in the marine algal species of the Mamiellales such as *Micromonas* and *Ostreococcus* is included with class I proteins (Additional file 1), this is likely serendipitous given the fact that each contains only one PABP gene.

### The first class III PABP appears in lycophytes

As in *P. patens*, four PABP genes are present in the lycophyte *Selaginella moellendorffii* genome. However, only three of these (*S. moellendorffii* 269256, 21095, and 101161) cluster with class I members and the fourth (*S. moellendorffii* 74731) clusters with class III proteins (Figure 3 and Additional file 1). This suggests that divergence within the PABP gene family had begun by the appearance of lycophytes. To investigate this possibility further, the introns of *P. patens* and *S. moellendorffii* PABP genes were examined. All four *P. patens* PABP genes share the same eight introns as do three of the *S. moellendorffii* PABP genes (Figure 4). The fourth *S. moellendorffii* gene, however, contains only two introns conserved with the other three *S. moellendorffii* genes and the four *P. patens* genes which are also the only two introns conserved with *C. reinhardtii*. This fourth *S. moellendorffii* gene (*S. moellendorffii* 74731) has six introns not present in the other *S. moellendorffii* or *P. patens* genes (Figure 4). Thus, the phylogenetic analysis and intron data indicate that this *S. moellendorffii* gene has diverged in gene structure as well as in protein sequence. The introns present in *S. moellendorffii* 74731 are nearly identical to those in the class III PAB7 gene of *A. thaliana* (Figure 4), indicating that a class III PABP gene first appeared in *S. moellendorffii*.

The genome sequence of any fern or fern ally has not been reported and only partial PABP sequences are available so the size of the PABP gene family in these species is unknown. However, available partial PABP sequences cluster with either class I or class III PABP proteins, similar to the genes in *S. moellendorffii* (Figure 3 and Additional file 1), suggesting that the PABP gene family had not substantially diverged beyond the family in lycophytes.

### PAB1 is not an orphan gene but a recently evolved class I PABP gene

The genome sequence of every species examined contains at least one class I gene (Figure 3 and Additional file 1), indicating that class I proteins have been maintained throughout land plant evolution. Two homologs are present in the gymnosperm species *Picea abies* for which the genome sequence was recently reported [48] (Figure 3 and Additional file 1). Homologs are also present in other gymnosperm species such as *Cycas micholitz* and *Sundacarpus amarus* (Figure 3 and Additional file 1). In flowering plant species, one class I protein is typically present which is most similar to *A. thaliana* PAB5 although some dicot species also contain related genes which are likely paralogs to *A. thaliana* PAB5. The first of these is represented by *A. thaliana* PAB3. Homologs of PAB3 are present only in *Arabidopsis lyrata*, *Capsella rubella*, and *Brassica rapa* (Figure 3 and Additional file 1), suggesting that PAB3 is a recent duplication of PAB5 in the Brassicaceae.

**Figure 5:**
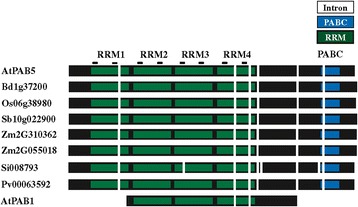
**Monocot class I PABP proteins are PAB5-like homologs.** Comparison of the intron positions of monocot class I PABP genes with *A. thaliana* PAB5 (At1g71770) and PAB1 (At1g34140). RRMs (green), PABC domain (blue), RNP1 and RNP2 motifs (lines), and introns (vertical white bars) are indicated. Monocot protein sequences used were: *Brachypodium distachyon* Bd1g37200; *Oryza sativa* Os06g38980; *Sorghum bicolor* Sb10g022900; *Zea mays* Zm2G310362 and Zm2G055018; *Setaria italica* Si008793; and *Panicum virgatum* Pv00063592.

The second example is represented by *A. thaliana* PAB1 which is confined to *A. lyrata*, *C. rubella*, and *Thellungiella halophila* but is not present in *B. rapa* (Figure 3 and Additional file 1). The phylogenetic analysis suggests that PAB1 is not an orphan gene or the sole representative of class IV PABP genes as previously reported [35] but rather it likely arose from a recent duplication of PAB5 or PAB3 during the evolution of the Brassicaceae. The phylogenetic analysis includes monocot class I genes as a group lying outside the PAB5/PAB3/PAB1 cluster although this is not well supported by the bootstrap value. To determine their relationship within the class I cluster, the monocot class I gene structure was compared to PAB1 and PAB5 from *A. thaliana*. Class I monocot sequences contain the RRM1 and PABC domain which is lacking in PAB1 (Figure 5). Additionally, examination of the gene structure revealed that class I monocot sequences share the same introns as *A. thaliana* PAB5 but not *A. thaliana* PAB1 (Figure 5). The conservation of domains and introns argue that the monocot class I genes are PAB5 orthologs and not PAB1 orthologs, consistent with the conclusion that the PAB1 cluster arose from a recent duplication of PAB3 or PAB5 within the Brassicaceae.

### The foundational class III PABP is maintained during land plant evolution

**Figure 6:**
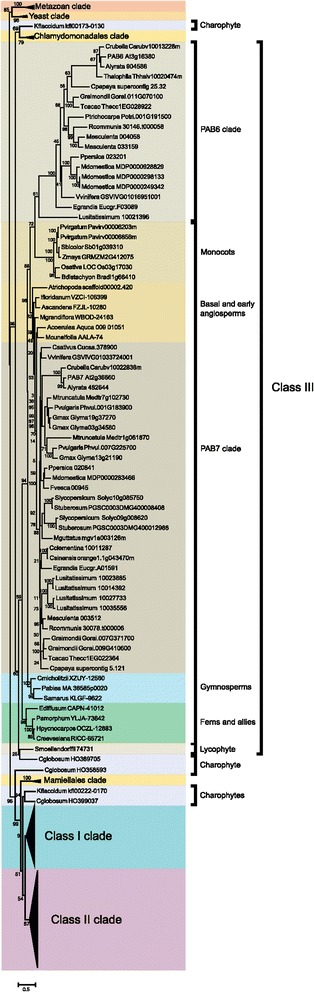
**Phylogenetic analysis of class III PABP proteins in plants.** A phylogenetic tree of class III PABP proteins in plants was constructed using the maximum-likelihood method. Numbers on each branch denote percentages of bootstrap support.

Following the appearance of the first class III gene in *S. moellendorffii* (74731), class III genes are retained nearly throughout subsequent land plant evolution (Figure 6). One partial sequence from *C. globosum* (HO369705) clustered with *S. moellendorffii* 74731 but as the *C. globosum* sequence is only a partial EST, it is not possible to draw a definitive conclusion from this observation. However, the fact that all four of the *P. patens* genes are highly similar to one another at the amino acid level, that they all cluster with class I genes, and their introns are unlike those of class III genes (Figure 4), it is unlikely that fresh water algae contained a class III gene that was then lost during the evolution of *P. patens* only to reappear in *S. moellendorffii*. It is more likely that this *C. globosum* partial sequence represents a region that serendipitously shares more similarity with class III genes than class I genes.

Most species contain one class III gene that is most similar to *A. thaliana* PAB7 although some species contain more than one PAB7-like gene, such as *Gossypium raimondii*, *Solanum lycopersicum*, and *Solanum tuberosum*, each of which contain two, *Glycine max* which has three, and *Linum usitatissimum* which has four (Figure 6). Interestingly, a few species lack a PAB7-like gene, including *B. rapa*, *T. halophila*, and *Populus trichocarpa*. Given how early this class III gene arose and its presence in most of the subsequent evolution of land plant, it is intriguing that select species have lost this class III gene member.

### The PAB6-like class III member arose during rosid evolution

**Figure 7:**
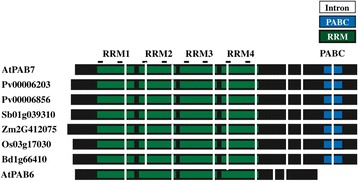
**Monocot class III PABP proteins are PAB7-like homologs.** Comparison of the intron positions of monocot class III PABP genes with *A. thaliana* PAB7 (At2g36660) and PAB6 (At3g16380). RRMs (green), PABC domain (blue), RNP1 and RNP2 motifs (lines), and introns (vertical white bars) are indicated. Monocot protein sequences used were: *B. distachyon* Bd1g66410; *O. sativa* Os03g17030; *S. bicolor* Sb01g039310; *Z. mays* Zm2G412075; and *P. virgatum* Pv00006856 and Pv00006203.

The other class III gene member present in some plant species is represented by *A. thaliana* PAB6. It is distinct from the PAB7 members in that it lacks the PABC domain, which serves as a characteristic feature of PAB6 homologs. No PAB6 homolog is present in the asterid species examined but PAB6 homologs are present in *Vitis vinifera* and most rosids with several notable exceptions, including *Medicago truncatula*, *Phaseolus vulgaris*, *Glycine max, Cucumis sativus*, *Fragaria vesca*, *B. rapa*, and *Citrus* species (Figure 6). Although the phylogenetic analysis includes monocot class III genes in the PAB6-like clade, this is not well supported by the gene structure (Figure 7). Monocot class III proteins contain the full PABC domain which is lacking in PAB6 homologs (Figure 7). Additionally, the introns present in the monocot class III genes are similar to *A. thaliana* PAB7 but not to *A. thaliana* PAB6 (Figure 7). The conservation of domains and introns in monocots argue strongly that their class III genes are PAB7 orthologs. Moreover, the phylogenetic analysis suggests that PAB6 arose from a duplication of PAB7 during rosid evolution and not earlier.

### Class II PABP proteins appear in seed plants

**Figure 8:**
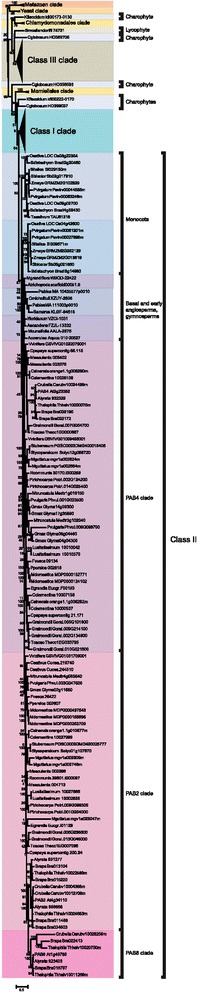
**Phylogenetic analysis of class II PABP proteins in plants.** A phylogenetic tree of class II PABP proteins in plants was constructed using the maximum-likelihood method. Numbers on each branch denote percentages of bootstrap support.

**Figure 9:**
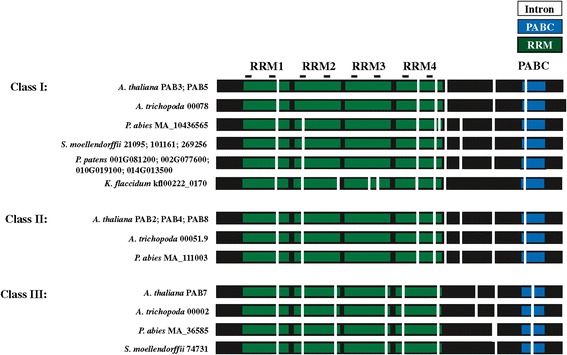
**Class I, class II, and class III PABP genes are present in gymnosperm and basal angiosperm species.** Comparison of the intron positions in PABP genes from the non-seed plant species *P. patens* and *S. moellendorffii*, the gymnosperm *P. abies*, the basal angiosperm *A. trichopoda,* and *A. thaliana*. RRMs (green), PABC domain (blue), RNP1 and RNP2 motifs (lines), and introns (vertical white bars) are indicated. Sequence for the *P. abies* class I gene was assembled from MA_10436565 and GQ03616_F15.1 whereas sequence for the *P. abies* class III gene was assembled from MA_36585 and UCPabies_isotig05724. Complete sequence for *P. abies* class I gene MA_18005 and class II gene MA_10435577 was not available. The *K. flaccidum* gene shown, the four *P. patens* genes, and three of four *S. moellendorffii* genes are progenitor PABP genes but are included with class I genes for comparison.

The *A. thaliana* genome contains three class II PABP genes, i.e., PAB2, PAB4, and PAB8, which are widely expressed, particularly in vegetative tissues [35,37,45]. Class II genes retain the introns of the progenitor gene which are distinct from those of class III genes (Figure 1). The introns of class I genes are similar to those in class II genes except in two cases where the introns have been lost from the progenitor gene and these differences can be used as identifiers of each gene class (Figure 1). By the evolution of gymnosperms such as *P. abies*, two class II members are present in *P. abies* which lie outside the PAB2, PAB4, or PAB8 subclades (Figure 8), suggesting that they predate the duplication events that occurred during the subsequent evolution of angiosperms. The introns in the *P. abies* class II member for which complete sequence information is available are virtually identical to those of class II genes (Figure 9), supporting the conclusion that this *P. abies* gene is a true class II gene. The *P. abies* genome also has two class I genes and one class III gene (Additional file 1).

By the evolution of *Amborella trichopoda*, which predated angiosperm diversification and therefore is the most recent common ancestor of all extant flowering plants [49] (see Additional file 2), one class II PABP gene is present in addition to the one class I and one class III gene (Figure 8 and Additional file 1). The introns in the *A. trichopoda* class II member are identical to those of *A. thaliana* class II genes as are the introns of the *A. trichopoda* class I and class III genes with those of *A. thaliana* class I and class III genes, respectively (Figure 9). A class II gene is present in other basal angiosperms and the gene in each basal angiosperm lies outside the PAB2, PAB4, or PAB8 subclades, suggesting that they predate the duplication events that occurred during subsequent angiosperm evolution.

Two or more class II genes are present in the monocot species examined and these are divided into two subgroups (Figure 8), suggesting gene duplication prior to speciation. Both of these class II subgroups lie outside the PAB2, PAB4, or PAB8 subclades, suggesting that the class II progenitor gene did not duplicate prior to the appearance of monocots. The presence of a single class II member in the *Aquilegia caerulea* genome that lies outside the PAB2, PAB4, or PAB8 subclades (Figure 8), supports the conclusion that the class II progenitor gene did not duplicate prior to the appearance of monocots and had not yet occurred by the appearance of *A. caerulea*. Class II PAB4-like and PAB2-like proteins are present in rosids such as *V. vinifera* and asterids such as *Solanum* species, suggesting that duplication of the class II progenitor gene had occurred by this point.

Further duplication of either PAB4-like or PAB2-like genes occurred in some species (Figure 8). For example, PAB8 is most similar to PAB2 (Figure 2 and Figure 8) and species containing class II PAB8-like genes are limited to *A. lyrata*, *T. halophila*, and *B. rapa* (Figure 8), suggesting that PAB8 is a recent duplication of PAB2 in the Brassicaceae. Together, these findings demonstrate that three of the eight members of the *A. thaliana* PABP gene family, i.e., PAB1, PAB3, and PAB8, recently evolved in the Brassicaceae while PAB6 appears in rosids but not in asterids. Only PAB5 and PAB7 are present nearly throughout land plant evolution while class II PABPs distinct from the progenitor PABP genes appeared in seed plants and expanded separately in gymnosperms and following the appearance of core eudicots.

### The members of the PABP gene family exhibit differences in RRM and PABC domains

The RRM domains of PABP exhibit a typical structure for this type of RNA-binding domain that is composed of a canonical RNA binding fold of four stranded antiparallel β-sheets that lie in front of two α-helices [50]. Of the four β-sheets, two central strands (where the β1strand corresponds to RNP2 and the β3 strand corresponds to RNP1) contact poly(A) RNA directly. The α-helices and the linker regions within the RRM are involved in interactions with proteins such as eIF4G [10,12-15,18-20]; eIF4B [10,14,16-18]; PABP-interacting protein (Paip) 1 [51]; Paip2 [52]; DAZL [53]; CFIA/Rna15p [41,43]; and yeast nuclear import and export factors [54,55].

**Figure 10:**
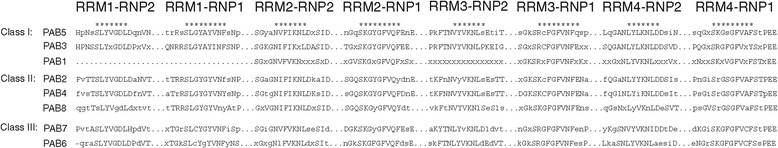
**Comparison of the conserved sequence for the RNP1 and RNP2 motifs within each RRM in class I, II, and III PABP genes.** Sequence logos for the region corresponding to the RNP1 and RNP2 motifs within each RRM of class I, II, and III PABP genes were generated from a sequence alignment of genes in higher plants using EBI MUSCLE. Following the alignment, the sequences were submitted to WebLogo Berkeley to generate a sequence logo. The consensus sequence corresponding to each *A. thaliana* PABP protein is shown with the RNP1 and RNP2 motifs indicated with asterisks.

To compare the RNP1 and RNP2 motifs for each type of PABP present in *A. thaliana*, a graphical logo showing the conservation of sequence at each residue was generated from a sequence alignment of PABP orthologs from the rosid species that were used in our phylogenetic analysis and a consensus for each type was derived. The sequence comprising the RNP1 and RNP2 motifs were largely conserved including the hydrophobic residues known to make contact with RNA (Figure 10). The most significant difference was the lack of an RRM1 for PAB1-like proteins. As the first two RRMs are thought to contribute most to poly(A) binding [3,50,56], this may affect the strength or type of RNAs bound by this member of the family. As eIF4G, eIFiso4G, and eIF4B also interact within RRM1, the absence of this domain in PAB1-like proteins would be expected to abolish the interaction with these partners. No consensus could be generated from the RNP2 motif in RRM3 of PAB1-like proteins as there was some variation in sequence in the very few species that contain this PABP isoform and this variation may affect the strength or type of RNAs bound by this member of the family.

**Figure 11:**
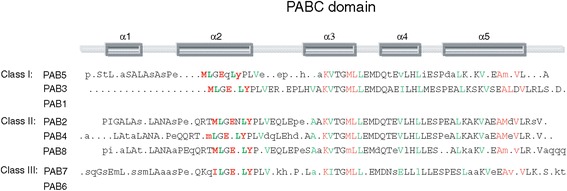
**Comparison of the conserved sequence for the PABC domain in class I, II, and III PABP genes.** Sequence logos for the PABC domain of class I, II, and III PABP genes were generated from a sequence alignment of genes in higher plants using EBI MUSCLE. Following the alignment, the sequences were submitted to WebLogo Berkeley to generate a sequence logo. Residues involved in binding PAM2 motif-containing proteins are indicated in red whereas those involved in building the hydrophobic core of the PABC domain are indicated in green. The α-helices of the domain are indicated above the sequence.

The plant PABC domain contains five α-helices that adopt a right-handed super coil fold [57]. The PABC domain interacts with PAM2 motif-containing proteins such as Paip1 [51]; Paip2 [52]; eukaryotic release factor 3 (eRF3) [58,59]; ERD/PCI6 from *C. sativus* which down regulates PABP-dependent translation [60], zucchini mosaic potyvirus RNA-dependent RNA polymerase [61], and other PAM2 motif-containing proteins [57]. Because of its function in protein interactions, a similar sequence logo was generated for the PABC domain from orthologs of rosid species used in our phylogenetic analysis. From each logo, a consensus was derived for each type of PABP. For those PABP types that contain a PABC domain, most of the critical residues within the domain were conserved and present in α helices 2, 3, and 5, including those involved in building the hydrophobic core of the PABC domain and those involved in binding PAM2 motif-containing proteins (Figure 11) [62]. The most significant difference among the PABP proteins is the lack of this domain in PAB1 and PAB6 which would render them incapable of interacting with PAM2 motif-containing proteins (Figure 11).

## Discussion

### Expansion of the PABP gene family predates land colonization

**Figure 12:**
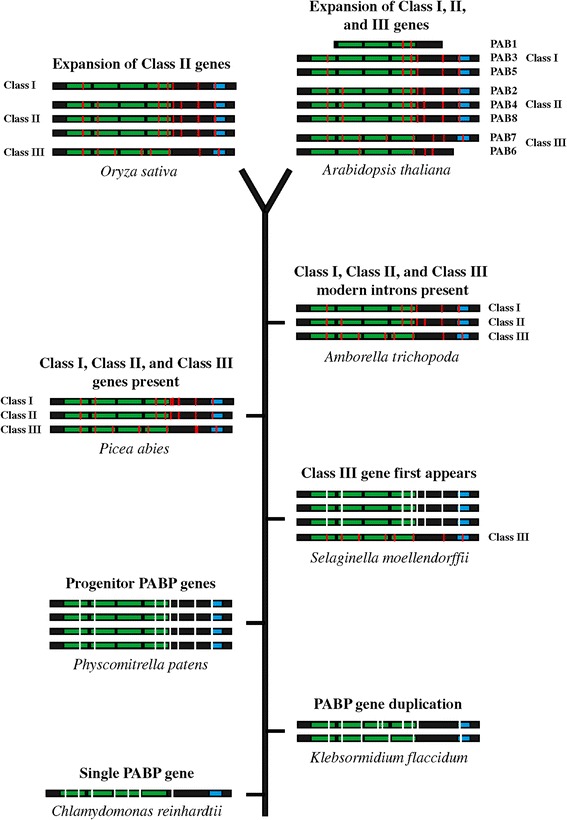
**Timeline of appearance of class I, II, and III PABP genes during land plant evolution.** The PABP genes of marine algae (*C. reinhardtii*), fresh water algae (*K. flaccidum*), the non-seed plant species *P. patens* and *S. moellendorffii*, the gymnosperm *P. abies*, the basal angiosperm *A. trichopoda,* the monocot *O. sativa* (rice), and *A. thaliana* are shown along a timeline of plant evolution from bottom to top. RRMs (green) and PABC domain (blue) are indicated. Vertical white bars represent introns in progenitor PABP genes whereas vertical red bars indicate introns in PABP genes upon appearance of specific class members. The number of genes represents the size of the PABP gene family for each species shown except *P. abies* where only the class I (MA_10436565), class II (MA_111003), and class III (MA_36585) genes are shown.

The results presented here demonstrate that the PABP gene family expanded from a single gene in marine algae to two genes in fresh water algae to a four member gene family upon the appearance of non-vascular plants. The four similar genes in *P. patens* subsequently diverged into the three classes of PABP genes present in higher plants today. That one of the *K. flaccidum* PABP genes contained six of the nine introns conserved in higher plants and three introns conserved in *C. reinhardtii* PABP suggests that it was in transition from the gene structure present in marine algae to that observed for higher plant PABP genes and that several of the introns characteristic of higher plants had evolved prior to colonization of land. The four members of the *P. patens* PABP gene family represent two pairs of highly similar genes, indicating duplication of an earlier two member gene family, consistent with the presence of a two gene family present in *K. flaccidum* (Figure 12). The high degree of similarity among the four *P. patens* PABP proteins, however, suggests that they likely participate in similar pathways and functions.

### The first class III member appears in vascular plants

By the appearance of *S. moellendorffii*, however, one of the four members of the PABP gene family had diverged in protein primary structure and in gene structure to become the first class III PABP protein to appear during plant evolution (Figure 12). This conclusion is supported by the phylogenetic analysis that includes this *S. moellendorffii* PABP protein within the class III clade and by its intron structure which is characteristic of class III PAB7-like homologs observed throughout subsequent plant evolution. This suggests that divergence within the PABP gene family had begun by the appearance of lycophytes. Therefore, of the four progenitor *P. patens* genes, one likely evolved into the class III gene present in *S. moellendorffii* while the other three remained as progenitor genes.

Following their appearance, class III genes are retained throughout subsequent plant evolution, suggesting that a PAB7-like function was necessary during early land plant evolution and was maintained thereafter. The class III gene PAB6 is most highly similar to PAB7 but unlike the latter, PAB6 evolution is relatively recent, appearing in rosids but not in asterid species. PAB6 differs from PAB7 in that it is missing the highly conserved PABC domain that is involved in protein interactions. How this affects the function of PAB6 is presently unknown, but the ability to bind poly(A) RNA while unable to interact with PAM2 motif-containing proteins may alter normal PABP function substantially. Although PAB6 lacks the PABC domain, it contains some sequence downstream of the four RRM domains. This sequence is not well conserved among those rosid species containing a PAB6 ortholog. For example, although this region is reasonably conserved among *A. thaliana*, *A. lyrata*, *C. rubella*, and *T. halophila*, it is not well conserved with other rosid PAB6 orthologs (data not shown). This indicates that PAB6 is undergoing dynamic evolution. Despite the phylogenetic grouping of monocot class III genes with PAB6, they are in fact PAB7 orthologs based on their intron pattern and the presence of an intact PABC domain. The absence of PAB6 homologs in basal angiosperm species or monocots supports the conclusion that PAB6 evolved following the appearance of dicots (Figure 12).

Phylogenetic analysis included the four progenitor *P. patens* PABP genes within the class I clade as it did three of the four *S. moellendorffii* PABP genes and sequences from ferns and fern allies. The bootstrap support for these, however, was low and these sequences are nearly as similar to class II genes. In contrast to the phylogenetic analysis, class II PABP genes retain all introns of the PABP progenitor genes observed in *P. patens* whereas class I genes retain all but two introns of the progenitor genes. This could be interpreted as class II genes being basal to class I genes where the latter evolved from the former as previously proposed [35]. Given the presence of four highly similar progenitor PABP genes in *P. patens*, however, the more likely explanation is that following evolution of one of the four progenitor PABP genes in *P. patens* into a class III PAB7-like gene in *S. moellendorffii*, class I and class II genes evolved independently from the remaining progenitor PABP genes. Class II genes retained all introns whereas class I genes lost two of the eight progenitor introns by the time *A. trichopoda* evolved (Figure 12). Interestingly, the gymnosperm class I gene of *P. abies* (MA 10436565) retains all eight introns of the progenitor gene (Figure 12), perhaps suggesting that the two introns lost in the *A. trichopoda* class I gene, in which the characteristic class I intron pattern is first observed, were lost just prior to the evolution of basal angiosperms.

### PAB1 and PAB3-like genes are recently evolved class I members

Phylogenetic analysis indicated class I genes in *A. thaliana* include PAB1, PAB3, and PAB5. Previously, PAB1 was designated an orphan gene and was proposed to be the sole representative of class IV PABP genes [35]. However, the phylogenetic analysis revealed that PAB1 is present in only a few species within the Brassicaceae, including *A. lyrata*, *C. rubella*, and *T. halophila* but not in *B. rapa*. Its similarity with PAB5 suggests that it is a class I gene that arose recently through duplication of PAB3 or PAB5. It is distinct from other class I genes in that it is missing the RRM1 and the PABC domain entirely. How this affects its function is presently unknown although it is possible that in the absence of its ability to interact with partner proteins may confer a very different function to the protein. Although the PAB1 ortholog of *T. halophila* lacks the PABC domain like other PAB1 orthologs, it does contain an RRM1, suggesting that the RRM1 was lost specifically in the *A. thaliana*, *A. lyrata*, *C. rubella* clade. This indicates that PAB1 is undergoing dynamic evolution following its recent appearance. No PAB1-like homolog is present in monocots as the monocot class I genes are PAB5 orthologs based on their intron pattern and the presence of an RRM1 as well as an intact PABC domain. As with PAB1, phylogenetic analysis demonstrated that PAB3 appeared in the Brassicaceae, indicating a recent duplication of PAB5 to which it shares the highest degree of similarity.

### Class I and class II genes evolved independently from progenitor PABP genes

As suggested above, class II genes likely evolved independently from progenitor PABP genes. *P. patens* contains four PABP progenitor genes whereas by the appearance of the basal angiosperm, *A. trichopoda*, the PABP gene family contains just three members, representing one each of the three gene classes (Figure 12). Therefore, each class likely evolved from three of the four original progenitor PABP genes while the fourth was lost subsequent to *S. moellendorffii*. Only a single class II gene is present in basal and early angiosperms up to and including the appearance of *A. caerulea*. The presence of two or more class II genes in *V. vinifera* and in *Solanum* species, however, suggests that duplication of the single class II gene had occurred following the evolution of basal eudicots which then evolved into the PAB2-like and PAB4-like subgroups of class II genes. As with PAB3, PAB8 appears only in the Brassicaceae, indicating it is a recent duplication of PAB2 to which it shares the highest degree of similarity.

The dynamic changes in the PABP gene family suggest the family is expanding and diverging with evolving needs. The evolution of the PABP gene family appears to be particularly rapid within the Brassicaceae where PAB 1, PAB3, and PAB8 have appeared specifically. Interestingly, not only do PAB3 and PAB8 appear in *B. rapa*, but this species has lost PAB6 and PAB7, while the PAB5 ortholog has expanded to three genes. These represent more changes in PABP gene family members than almost any other species examined. Such changes also raise the question of possible functional differences among the three classes which have not been examined in plants but which their differences in expression may reveal clues to.

### PABP class member expression specificity is maintained during plant evolution

Despite its early appearance in evolution, expression from the class III PAB7 gene is not observed by Northern analysis in *A. thaliana* [35]. However, its expression could be detected in siliques using RT-PCR [35]. *In silico* analysis in *A. thaliana* suggests high expression restricted to tricellular and mature pollen as well as pollen tubes following their growth through stigma and style tissue although much lower expression is also present in developing embryos (Arabidopsis eFP Browser; http://bar.utoronto.ca/efp/cgi-bin/efpWeb.cgi). Similar analysis of the PAB7 ortholog in maize indicates that its expression is restricted to anthers and is expressed during the mid to late stage of endosperm development but not expressed in embryos (MaizeGDB; http://www.maizegdb.org). It is this restricted expression that may explain why PAB7 expression was not observed in Northerns. Although most species contain just a single PAB7 homolog, some species contain multiple PAB7 homologs. Of the two PAB7 homologs in *Solanum lycopersicum* (Solyc09g008620 and Solyc10g085750), expression is restricted to flower buds and opened flowers (Tomato eFP Browser; http://bar.utoronto.ca/efp_tomato/cgi-bin/efpWeb.cgi). Of the three PAB7 homologs in *Glycine max*, the expression of two (GLYMA19g37270 and GLYMA 03 g34580) is restricted to flowers, pods, and root nodules (and the shoot apical meristem in the case of GLYMA 03 g34580) while the third (GLYMA19g37270) is not expressed in these tissues but rather in leaves and roots (http://bar.utoronto.ca/efp_soybean/cgi-bin/efpWeb.cgi). These data indicate that PAB7 expression is restricted largely to reproductive tissues in dicots and monocots but may be expressed in other tissues when additional paralogs are present. Like PAB7, PAB6 expression was not observed by Northern analysis in *A. thaliana* but was detected using RT-PCR in leaves and seedlings [35]. *In silico* analysis in *A. thaliana* indicates expression in leaves and, as with PAB7, in tricellular and mature pollen as well as in pollen tubes following their growth through stigma and style tissue (Arabidopsis eFP Browser).

Expression of the class I PAB5 gene in *A. thaliana* is observed in tapetum, pollen, ovules, and developing seeds whereas PAB3 expression is restricted to the tapetum and pollen [35,36]. *In silico* analysis indicates high expression of PAB5 and PAB3 throughout pollen development with much lower expression in pollen tubes following their growth through stigma and style tissue (Arabidopsis eFP Browser). Similar analysis of the PAB5 orthologs in maize (GRMZM2G310362 and GRMZM2G055018) indicates their expression is restricted to anthers (MaizeGDB) and in *Solanum lycopersicum* (Solyc00g009040), expression is restricted to flower buds and opened flowers (Tomato eFP Browser). Thus, like the class III PAB7 gene, class I PAB5 and PAB3 gene expression is restricted largely to reproductive tissues in dicots and monocots. In contrast, expression of PAB1 in *A. thaliana* was observed predominantly in roots [46] while *in silico* analysis indicates that, in addition to roots, PAB1 is expressed during late embryo development and in pollen (Arabidopsis eFP Browser).

Expression of class II genes occurs widely among tissues in *A. thaliana* with ESTs for PAB2 isolated from roots, seeds, whole plants, and flowers [35,45]. *In silico* analysis indicates high expression of PAB2 in virtually every organ throughout development with little change following biotic or abiotic stress, hormone treatment, or light cycle (Arabidopsis eFP Browser), suggesting constitutive expression in most tissues under most conditions. Virtually identical EST and expression patterns are observed for PAB4 and PAB8, raising the question of why the single class II gene present in basal and early angiosperm species underwent one or more duplications following the evolution of basal eudicots to result in multiple similar proteins with virtually identical expression patterns. It is possible that the expansion of the class II subclass of PABP genes resulted in PABP proteins with subtle differences in function, e.g., in protein or RNA interactions, or was needed to provide a higher level of class II protein expression that was not possible from a single gene.

### Divergence of PABP gene family members likely affects their protein interactions

The presence of three distinct classes of PABP proteins in higher plants raises the question of whether there may be class-specific functions. With the exception of PAB1, examination of the RNP motifs in each RRM responsible for contacting RNA did not reveal substantial differences among the three class types. The PABC domain of each class type was also substantially conserved except for PAB1 and PAB6 as they lack this domain. Sequence differences do exist, however, in other parts of the protein, e.g., the regions of the RRMs not directly involved in RNA binding and in the C-terminal region of the protein upstream of the PABC domain. Whether these regions may contribute to class-specific functional differences remains to be determined.

PAB1 and PAB6, the recently evolved and highly divergent members of class I and class III proteins, respectively, are most likely to exhibit the greatest functional differences from the other members of their individual classes as they lack the PABC domain and, in the case of PAB1, the RRM1 as well. As the first two RRMs of PABP are thought to contribute most to poly(A) binding [3,50,56], the absence of the RRM1 in PAB1 may affect the strength or type of RNAs it binds. Moreover, as eIF4G, eIFiso4G, and eIF4B bind within the RRM1, its absence in PAB1 would abolish these interactions. The PABC domain functions as another protein interacting region to which several proteins containing a PAM2 motif can bind such as Paip1 [51]; Paip2 [52]; eRF3 [58,59]. Several proteins in plants also interact with the PABC domain, including PCI6 and PCI243 of cucumber and Arabidopsis ERD15 (early-responsive to dehydration), each of which contains an N-terminal PAM2 motif [57,60,61]. ERD15 shares similarity with PCI6 and is similar to the mammalian Paip2 [52] in that it also is a small acidic protein. Other PAM2-containing proteins include Ataxin-2, Tob1/2, RBP37, RBP1, and CID3-CID13 [63,64]. Addition of PCI6 to wheat germ lysate or mouse ascites Krebs-2 lysate repressed translation suggesting that it may serve as a repressor of PABP function [60]. The absence of a PABC domain PAB1 and PAB6 might enable these proteins to escape the translational repression exerted by proteins such as PCI6, enabling translation to continue for those mRNAs to which PAB1 or PAB6 are bound.

On the other hand, PABP also interacts with the release factor, eRF3, which is required during translation termination [58,59,65]. The eRF3-PABP interaction enhances the efficiency of termination *in vivo* and mediates mRNA decay by regulating deadenylation in a translation-dependent manner [66]. Interaction of eRF3 with the PABC domain disrupts PABP multimerization [58]. Disruption of the eRF3-PABP interaction inhibits translation and the addition eRF3 to *in vitro* translation assays stimulates initiation [59]. Therefore, the absence of a PABC domain in PAB1 and PAB6 would render them unable to interact with eRF3 and if disruption of the eRF3-PABP interaction inhibits translation, the translation of those mRNAs binding PAB1 or PAB6 may be negatively affected. Because of the fundamental role that PABP plays in global gene expression, it will be intriguing to determine how the recent appearance of these PABP members, the recent appearance of PAB3 and PAB8, or the loss of PAB6 and PAB7 from species such as *B. rapa* might have been involved in the evolution of species in the Brassicaceae.

## Conclusions

Although PABP is encoded by a single gene in yeast and marine algae, the presence of two PABP genes in *K. flaccidum*, a fresh water algae, demonstrates expansion of the PABP gene family prior to the colonization of land. The first class III member appeared with the evolution of vascular plants and, together with class I genes, is expressed largely in reproductive tissues suggesting that supporting reproductive functions was an important early function of PABP. Class II vegetative-associated members, which first appear in gymnosperms, retain the introns of the progenitor genes. Two introns from a progenitor gene were lost during class I gene evolution but class I genes likely evolved independently of class II genes and not from a class II gene as previously suggested. By the appearance of the first known basal angiosperm, all three modern gene classes had fully evolved and the size of each gene class expanded further during subsequent evolution. This is especially the case in the Brassicaceae where three additional members appear: PAB1, a class I gene, PAB8, a class II gene, and PAB6, a class III gene. The recent evolution of PAB1 in the Brassicaceae demonstrates that this gene member did not evolve as an orphan gene directly from a PABP ancestral gene progenitor as was previously suggested but rather from an existing class I gene. The recent appearance of three new members of the PABP gene family also indicates that the family continues to undergo dynamic evolution.

## Methods

### Sequence alignment and phylogenetic analysis

The amino acid sequences of the *A. thaliana* PABP gene family were used to identify homologs in other species through BLAST searches of the NCBI [67], Phytozome v9.1 [68], the Amborella Genome Database [49], the Spruce Genome Project [48], or from the Onekp project (www.onekp.com). Reiterative searches of species were performed using PABP amino acid sequences from that species. Predicted protein sequences of genomic and ESTs were obtained using the ExPASy Translate tool [69]. Amino acid sequence alignments were performed by MAFFT [70]. Maximum-likelihood phylogenetic tree of PABP proteins was constructed using PhyML software (v3.1) [71] with 1000 bootstrap replicates and a default LG amino acid replacement matrix was used [72]. Numbers included on each branch denote percentages of bootstrap support.

Sequence logos for the region corresponding to the RNP1 and RNP2 motifs within each RRM of class I, II, and III PABP genes were generated from a sequence alignment of genes in higher plants using EBI MUSCLE (https://www.ebi.ac.uk/Tools/msa/muscle). Following the alignment, the sequences were submitted to WebLogo Berkeley (http://weblogo.berkeley.edu/logo.cgi) to generate a sequence logo. At each residue position the most conserved one, two, or sometimes three residues are shown as the consensus.

Gene sequences used the analyses were from *Arabidopsis thaliana* (At3g16380, At2g36660, At1g34140, At1g71770, At1g22760, At2g23350, At4g34110, At1g49760); *Arabidopsis lyrata* (904586, 482644, 313927, 339396, 335514, 888668, 931277, 923403, 932329); *Capsella rubella (*Carubv10013228m, Carubv10022838m, Carubv10011165m, Carubv10021938m, Carubv10011423m, Carubv10024499m, Carubv10004368m, Carubv10012708m, Carubv10028259m); *Thellungiella halophila (*Thhalv10020474m, Thhalv10009520m, Thhalv10018219m, Thhalv10024683m, Thhalv10000075m, Thhalv10022589m, Thhalv10020750m, Thhalv10011); *Brassica rapa* (Bra016118, Bra007984, Bra003915, Bra016371, Bra039195, Bra032172, Bra011488, Bra034603, Bra013104, Bra015220, Bra022413, Bra018797); *Carica papaya* (supercontig 5.121, supercontig 26.121, supercontig 55.115, supercontig 21.171, supercontig 200.24, supercontig 25.32); *Gossypium raimondii* (Gorai.011G070100, Gorai.009G410600, Gorai.007G371700, Gorai.007G054700, Gorai.005G101600, Gorai.009G214100, Gorai.002G134900, Gorai.010G021800, Gorai.013G046000, Gorai.005G236500); *Theobroma cacao* (Thecc1EG028922, Thecc1EG022364, Thecc1EG035855, Thecc1EG000667, Thecc1EG033795, Thecc1EG007096); *Populus trichocarpa* (Potri.001G191500, Potri.002G062700, Potri.005G198500, Potri.002G124200, Potri.014G025400, Potri.009G099300, Potri.001G304000); *Eucalyptus grandis* (Eucgr.F03089, Eucgr.A01591, Eucgr.F00193, Eucgr.I01129); *Citrus sinensis* (orange1.1g043470m, orange1.1g006641m, orange1.1g006290m, orange1.1g006282m, orange1.1g010577m); *Citrus clementina* (10011287, 10003530, 10025109, 10007158, 10000537, 10027999); *Cucumis sativus* (Cucsa.378900, Cucsa.364050, Cucsa.219740, Cucsa.244510); *Mimulus guttatus* (mgv1a003126m, mgv1a025142m, mgv1a002624m, mgv1a002664m, mgv1a023047m, mgv1a002809m, mgv1a002746m); *Manihot esculenta* (004058, 033159, 003512, M003279, 003402, 003376, 004713, 003396); *Ricinus communis* (30146.t000058, 30078.t000006, 28644.t000011, 30170.t000259, 29801.t000097); *Medicago truncatula* (Medtr7g102730, Medtr1g061870, AC233576 5, Medtr1g019150, Medtr3g102040, Medtr4g085540); *Prunis persica* (023201, 020841, 002584, 002618, 002607); *Malus domestica* (MDP0000928829, MDP0000298133, MDP0000249342, MDP0000283466, MDP0000152771, MDP0000124102, MDP0000497543, MDP0000156895, MDP0000253709, MDP0000224417); *Linum usitatissimum* (10021396, 10035556, 10027733, 10014392, 10023885, 10010042, 10010370, 10027886, 10002835); *Fragaria vesca* (00945, 27559, 09134, 26422); *Phaseolus vulgaris* (Phvul.001G183900, Phvul.007G225700, Phvul.007G084800, Phvul.001G023500, Phvul.008G098700, Phvul.003G047500); *Glycine max* (Glyma19g37270, Glyma03g34580, Glyma13g21190, Glyma20g31120, Glyma02g08480, Glyma14g09300, Glyma17g35890, Glyma06g04460, Glyma04g04300, Glyma02g11580); *Vitis vinifera* (GSVIVG01016951001, GSVIVG01033724001, GSVIVG01013699001, GSVIVG01022079001, GSVIVG01009455001, GSVIVG01031709001); *Solanum lycopersicum (*Solyc09g008620, Solyc10g085750, Solyc00g009040, Solyc12g088720, Solyc01g107870); *Sorghum bicolor* (Sb01g039310, Sb10g022900, Sb06g021850, Sb02g017910); *Solanum tuberosum* (PGSC0003DMG400012988, PGSC0003DMG400008408, PGSC0003DMG400025074, PGSC0003DMG400015406, PGSC0003DMG400025777); *Brachypodium distachyon* (Bradi1g66410, Bradi1g37200, Bradi3g20460, Bradi4g08430, Bradi5g14980); *Zea mays* (GRMZM2G412075, GRMZM2G310362, GRMZM2G055018, GRMZM2G102829, GRMZM2G352129, GRMZM2G013619); *Oryza sativa* (LOC Os03g17030, LOC Os06g38980, LOC Os08g22354, LOC Os09g02700, LOC Os04g42600); *Panicum virgatum* (Pavirv00006203m, Pavirv00006856m, Pavirv00063592m, Pavirv00044555m, Pavirv00063246m, Pavirv00061201m, Pavirv00027898m); *Setaria italica* (Si008793m, Si029150m, Si009571m); *Triticum aestivum* (TAU81318); *Magnolia grandiflora* (WBOD-24163, WBOD-22368, WBOD-22422); *Meliosma cuneifolia* (AALA-74, AALA-72819, AALA-2575); *Aquilegia coerulea* (Aquca 009 01051, Aquca 014 00454, Aquca 114 00019, Aquca 010 00627); *Illicium floridanum* (VZCI-106399, VZCI-6757, VZCI-1031); *Austrobaileya scandens* (FZJL-10280, FZJL-7789, FZJL-13332); *Amborella trichopoda* (scaffold00002.420, scaffold00051.9, scaffold00078.126); *Picea abies* (MA 36585p0020, MA 18005p0010, MA 10436565p0010, MA 10435577p0010, MA 111003p0010); *Cycas micholitz* (XZUY-12560, XZUY-2817, XZUY-2606); *Sundacarpus amarus* (KLGF-9622, KLGF-5882, KLGF-94618); *Equisetum diffusum* (CAPN-41012, CAPN-5595, CAPN-6199); *Polypodium amorphum* (YLJA-73642, YLJA-16058, YLJA-73494, YLJA-5966); *Homalosorus pycnocarpos* (OCZL-12883, OCZL-59433, OCZL-2016, OCZL-11876); *Cystopteris reevesiana* (RICC-65721, RICC-327, RICC-6181, RICC-65698, RICC-65784); *Selaginella moellendorffii* (74731, 21095, 101161, 269256); *Physcomitrella patens* (010G019100, 014G013500, 002G077600, 001G081200), *Klebsormidium flaccidum* (kfl00222_0170, kfl00173_0130); *Chaetosphaeridium globosum* (HO369705, HO358593, HO399037); *Volvox carteri* (Vocar20009857m); *Chlamydomonas reinhardtii* (Cre01.g039300); *Ostreococcus lucimarimus* (Chr 70395 50098); *Micromonas pusilla* (CCMP1545, RCC299); *Coccomyxa subellipsoidea* (C 40263); *Schizosaccharomyces pombe* (NM 001018809); *Saccharomyces cerevisiae* (NM 001179055); and *Homo sapiens* (PABEU190483, BC065540, NM 002568, NM 030979).

## Additional files

Additional file 1:
**Phylogenetic analysis of PABP proteins in plants.** A phylogenetic tree of PABP proteins in plants was constructed using the maximum-likelihood method. Numbers on each branch denote percentages of bootstrap support. PABP sequences from yeast and humans were used to root the tree. Additional file 2:
**Phylogenetic relationships among the plant species used in this study.** Plant groups and their relationships are shown to the left with the corresponding species included in the analysis listed to the right.

## Abbreviations

eIF: Translation initiation factor
PABP: Poly(A) binding protein
RRM: RNA recognition motif

## References

[1] Baker EJ, Belasco JG, Brawerman G (1993). Control of Poly(A) Length. Control of Messenger RNA Stability.

[2] Sachs AB, Davis RW, Kornberg RD (1987). A single domain of yeast poly(A)-binding protein is necessary and sufficient for RNA binding and cell viability. Mol Cell Biol.

[3] Kuhn U, Pieler T (1996). Xenopus poly(A) binding protein: functional domains in RNA binding and protein-protein interaction. J Mol Biol.

[4] Melo EO, Dhalia R, Martins de Sa C, Standart N, de Melo Neto OP (2003). Identification of a C-terminal poly(A)-binding protein (PABP)-PABP interaction domain: role in cooperative binding to poly (A) and efficient cap distal translational repression. J Biol Chem.

[5] Simón E, Séraphin B (2007). A specific role for the C-terminal region of the poly(A)-binding protein in mRNA decay. Nucleic Acids Res.

[6] Baer BW, Kornberg RD (1980). Repeating structure of cytoplasmic poly(A)-ribonucleoprotein. Proc Natl Acad Sci U S A.

[7] Ray BK, Lawson TG, Kramer JC, Cladaras MH, Grifo JA, Abramson RD, Merrick WC, Thach RE (1985). ATP-dependent unwinding of messenger RNA structure by eukaryotic initiation factors. J Biol Chem.

[8] Gallie DR (1991). The cap and poly(A) tail function synergistically to regulate mRNA translational efficiency. Genes Dev.

[9] Tarun SZ, Sachs AB (1996). Association of the yeast poly(A) tail binding protein with translation initiation factor eIF-4G. EMBO J.

[10] Le H, Tanguay RL, Balasta ML, Wei C-C, Browning KS, Metz AM, Goss DJ, Gallie DR (1997). Translation initiation factors eIF-iso4G and eIF-4B interact with the poly(A)-binding protein and increase its RNA binding activity. J Biol Chem.

[11] Wells SE, Hillner PE, Vale RD, Sachs AB (1998). Circularization of mRNA by eukaryotic translation initiation factors. Mol Cell.

[12] Piron M, Vende P, Cohen J, Poncet D (1998). Rotavirus RNA-binding protein NSP3 interacts with eIF4GI and evicts the poly(A) binding protein from eIF4F. EMBO J.

[13] Imataka H, Gradi A, Sonenberg N (1998). A newly identified N-terminal amino acid sequence of human eIF4G binds poly(A)-binding protein and functions in poly(A)-dependent translation. EMBO J.

[14] Le H, Browning KS, Gallie DR (2000). The phosphorylation state of poly(A)-binding protein specifies its binding to poly(A) RNA and its interaction with eukaryotic initiation factor (eIF) 4 F, eIFiso4F, and eIF4B. J Biol Chem.

[15] Fraser CS, Pain VW, Morley SJ (1999). The association of initiation factor 4 F with poly(A)-binding protein is enhanced in serum-stimulated Xenopus kidney cells. J Biol Chem.

[16] Bushell M, Wood W, Carpenter G, Pain VM, Morley SJ, Clemens MJ (2001). Disruption of the interaction of mammalian protein synthesis initiation factor 4B with the poly(A) binding protein by caspase- and viral protease-mediated cleavages. J Biol Chem.

[17] Cheng S, Gallie DR (2006). Wheat eukaryotic initiation factor 4B organizes assembly of RNA and eIFiso4G, eIF4A, and poly(A)-binding protein. J Biol Chem.

[18] Cheng S, Gallie DR (2007). eIF4G, eIFiso4G, and eIF4B bind the poly(A)-binding protein through overlapping sites within the RNA recognition motif domains. J Biol Chem.

[19] Cheng S, Gallie DR (2010). Competitive and noncompetitive binding of eIF4B, eIF4A, and the poly(A) binding protein to wheat translation initiation factor eIFiso4G. Biochemistry.

[20] Cheng S, Gallie DR (2013). Eukaryotic initiation factor 4B and the poly(A)-binding protein bind eIF4G competitively. Translat.

[21] Kessler SH, Sachs AB (1998). RNA recognition motif 2 of yeast Pab1p is required for its functional interaction with eukaryotic translation initiation factor 4G. Mol Cell Biol.

[22] Otero LJ, Ashe MP, Sachs AB (1999). The yeast poly(A)-binding protein Pab1p stimulates *in vitro* poly(A)-dependent and cap-dependent translation by distinct mechanisms. EMBO J.

[23] Kopeina GS, Afonina ZA, Gromova KV, Shirokov VA, Vasiliev VD, Spirin AS (2008). Step-wise formation of eukaryotic double-row polyribosomes and circular translation of polysomal mRNA. Nucleic Acids Res.

[24] Madin K, Sawasaki T, Kamura N, Takai K, Ogasawara T, Yazaki K, Takei T, Miura K, Endo Y (2004). Formation of circular polyribosomes in wheat germ cell-free protein synthesis system. FEBS Lett.

[25] Brandt F, Carlson LA, Hartl FU, Baumeister W, Grünewald K (2010). The three-dimensional organization of polyribosomes in intact human cells. Mol Cell.

[26] Mangus DA, Evans MC, Jacobson A (2003). Poly(A)-binding proteins: multifunctional scaffolds for the post-transcriptional control of gene expression. Genome Biol.

[27] Caponigro G, Parker R (1995). Multiple functions for the poly(A) binding protein in mRNA decapping and deadenylation in yeast. Genes Dev.

[28] Wilusz CJ, Gao M, Jones CL, Wilusz J, Peltz SW (2001). Poly(A)-binding proteins regulate both mRNA deadenylation and decapping in yeast cytoplasmic extracts. RNA.

[29] Bernstein P, Peltz SW, Ross J (1989). The poly(A)-poly(A)-binding protein complex is a major determinant of mRNA stability in vitro. Mol Cell Biol.

[30] Ford L, Bagga PS, Wilusz J (1997). The poly(A) tail inhibits the assembly of a 3′-to-5′ exonuclease in an in vitro RNA stability system. Mol Cell Biol.

[31] Wormington M, Searfoss AM, Hurney CA (1996). Overexpression of poly(A) binding protein prevents maturation-specific deadenylation and translational inactivation in *Xenopus* oocytes. EMBO J.

[32] Schwartz DC, Parker R: **Interaction of mRNA Translation and mRNA Degradation in*****Saccharomyces Cerevisiae***. In *Translational Control of Gene Expression.* Edited by Sonenberg N, *et al*. Cold Spring Harbor, NY: Cold Spring Harbor Laboratory Press; 2000:807–825.

[33] Gao M, Wilusz CJ, Peltz SW, Wilusz J (2001). A novel mRNA decapping activity in HeLa cytoplasmic extracts is regulated by AU-rich elements. EMBO J.

[34] Lemay JF, D’Amours A, Lemieux C, Lackner DH, St-Sauveur VG, Bähler J, Bachand F (2010). The nuclear poly(A)-binding protein interacts with the exosome to promote synthesis of noncoding small nucleolar RNAs. Mol Cell.

[35] Belostotsky DA (2003). Unexpected complexity of poly(A)-binding protein gene families in flowering plants: three conserved lineages that are at least 200 million years old and possible auto-cross-regulation. Genetics.

[36] Belostotsky DA, Meagher RB (1996). A pollen-, ovule-, and early embryo-specific poly(A) binding protein from Arabidopsis complements essential functions in yeast. Plant Cell.

[37] Palanivelu R, Belostotsky DA, Meagher RB (2000). Conserved expression of Arabidopsis thaliana poly (A) binding protein 2 (PAB2) in distinct vegetative and reproductive tissues. Plant J.

[38] Chekanova JA, Shaw RJ, Belostotsky DA (2001). Analysis of an essential requirement for the poly(A) binding protein function using cross-species complementation. Curr Biol.

[39] Hammell CM, Gross S, Zenklusen D, Heath CV, Stutz F, Moore C, Cole CN (2002). Coupling of termination, 3′processing, and mRNA export. Mol Cell Biol.

[40] Thakurta AG, Yoon J, Dhar R (2002). *Schizosaccharomyces pombe* spPABP, a homologue of *Saccharomyces cerevisiae* Pab1p, is a non-essential, shuttling protein that facilitates mRNA export. Yeast.

[41] Minvielle-Sebastia L, Preker PJ, Wiederkehr T, Strahm Y, Keller W (1997). The major yeast poly(A)-binding protein is associated with cleavage factor IA and functions in premessenger RNA 3¢-end formation. Proc Natl Acad Sci U S A.

[42] Chekanova JA, Belostotsky DA (2003). Evidence that poly(A) binding protein has an evolutionarily conserved function in facilitating mRNA biogenesis and export. RNA.

[43] Amrani N, Minet M, Le Gouar M, Lacroute F, Wyers F (1997). Yeast Pab1 interacts with Rna15 and participates in the control of the poly(A) tail length in vitro. Mol Cell Biol.

[44] Le H, Gallie DR (2000). Sequence diversity and conservation of the poly(A)-binding protein in plants. Plant Sci.

[45] Hilson P, Carroll KL, Masson PH (1993). Molecular characterization of PAB2, a member of the multigene family coding for poly(A)-binding proteins in Arabidopsis thaliana. Plant Physiol.

[46] Belostotsky DA, Meagher RB (1993). Differential organ-specific expression of three poly(A)-binding protein genes from *Arabidopsis thaliana*. Proc Natl Acad Sci U S A.

[47] Hori K, Maruyama F, Fujisawa T, Togashi T, Yamamoto N, Seo M, Sato S, Yamada T, Mori H, Tajima N, Moriyama T, Ikeuchi M, Watanabe M, Wada H, Kobayashi K, Saito M, Masuda T, Sasaki-Sekimoto Y, Mashiguchi K, Awai K, Shimojima M, Masuda S, Iwai M, Nobusawa T, Narise T, Kondo S, Saito H, Sato R, Murakawa M, Ihara Y (2014). *Klebsormidium flaccidum* genome reveals primary factors for plant terrestrial adaptation. Nat Commun.

[48] Nystedt B, Street NR, Wetterbom A, Zuccolo A, Lin YC, Scofield DG, Vezzi F, Delhomme N, Giacomello S, Alexeyenko A, Vicedomini R, Sahlin K, Sherwood E, Elfstrand M, Gramzow L, Holmberg K, Hällman J, Keech O, Klasson L, Koriabine M, Kucukoglu M, Käller M, Luthman J, Lysholm F, Niittylä T, Olson A, Rilakovic N, Ritland C, Rosselló JA, Sena J (2013). The Norway spruce genome sequence and conifer genome evolution. Nature.

[49] Amborella Genome Project (2013). The Amborella genome and the evolution of flowering plants. Science.

[50] Deo RC, Bonanno JB, Sonenberg N, Burley SK (1999). Recognition of polyadenylate RNA by the poly(A)-binding protein. Cell.

[51] Roy G, De Crescenzo G, Khaleghpour K, Kahvejian A, O’Connor-McCourt M, Sonenberg N (2002). Paip1 interacts with poly(A) binding protein through two independent binding motifs. Mol Cell Biol.

[52] Khaleghpour K, Svitkin YV, Craig AW, DeMaria CT, Deo RC, Burley SK, Sonenberg N (2001). Translational repression by a novel partner of human poly(A) binding protein, Paip2. Mol Cell.

[53] Collier B, Gorgoni B, Loveridge C, Cooke HJ, Gray NK (2005). The DAZL family proteins are PABP-binding proteins that regulate translation in germ cells. EMBO J.

[54] Brune C, Munchel SE, Fischer N, Podtelejnikov AV, Weis K (2005). Yeast poly(A)-binding protein Pab1 shuttles between the nucleus and the cytoplasm and functions in mRNA export. RNA.

[55] Dunn EF, Hammell CM, Hodge CA, Cole CN (2005). Yeast poly(A)-binding protein, Pab1, and PAN, a poly(A) nuclease complex recruited by Pab1, connect mRNA biogenesis to export. Genes Dev.

[56] Burd CG, Matunis EL, Dreyfuss G (1991). The multiple RNA binding domains of the mRNA poly(A)-binding protein have different RNA-binding activities. Mol Cell Biol.

[57] Siddiqui N, Osborne MJ, Gallie DR, Gehring K (2007). Solution structure of the PABC domain from wheat poly (A)-binding protein: an insight into RNA metabolic and translational control in plants. Biochemistry.

[58] Hoshino S, Hosoda N, Araki Y, Kobayashi T, Uchida N, Funakoshi Y, Katada T (1999). Novel function of the eukaryotic polypeptide-chain releasing factor 3 (eRF3/GSPT) in the mRNA degradation pathway. Biochemistry.

[59] Uchida N, Hoshino S, Imataka H, Sonenberg N, Katada T (2002). A novel role of the mammalian GSPT/eRF3 associating with poly(A)-binding protein in cap/poly(A)-dependent translation. J Biol Chem.

[60] Wang X, Grumet R (2004). Identification and characterization of proteins that interact with the carboxy terminus of poly(A)-binding protein and inhibit translation in vitro. Plant Mol Biol.

[61] Wang X, Ullah Z, Grumet R (2000). Interaction between zucchini yellow mosaic potyvirus RNA-dependent RNA polymerase and host poly-(A) binding protein. Virology.

[62] Kozlov G, Trempe JF, Khaleghpour K, Kahvejian A, Ekiel I, Gehring K (2001). Structure and function of the C-terminal PABC domain of human poly(A)-binding protein. Proc Natl Acad Sci U S A.

[63] Albrecht M, Lengauer T (2004). Survey on the PABC recognition motif PAM2. Biochem Biophys Res Commun.

[64] Bravo J, Aguilar_Henonin L, Olmedo G, Guzman P (2005). Four distinct classes of proteins as interaction partners of the PABC domain of Arabidopsis thaliana poly(A)-binding proteins. Mol Genet Genomics.

[65] Cosson B, Couturier A, Chabelskaya S, Kiktev D, Inge-Vechtomov S, Philippe M, Zhouravleva G (2001). Poly(A)-binding protein acts in translation termination via eukaryotic release factor 3 interaction and does not influence [PSI(+)] propagation. Mol Cell Biol.

[66] Hosoda N, Kobayashi T, Uchida N, Funakoshi Y, Kikuchi Y, Hoshino S, Katada T (2003). Translation termination factor eRF3 mediates mRNA decay through the regulation of deadenylation. J Biol Chem.

[67] Altschul SF, Wootton JC, Zaslavsky E, Yu YK (2010). The construction and use of log-odds substitution scores multiple sequence alignment. PLoS Comput Biol.

[68] Goodstein DM, Shu S, Howson R, Neupane R, Hayes RD, Fazo J, Mitros T, Dirks W, Hellsten U, Putnam N, Rokhsar DS (2012). Phytozome: a comparative platform for green plant genomics. Nucleic Acid Res.

[69] Gasteiger E, Gattiker A, Hoogland C, Ivanyi I, Appel RD, Bairoch A (2003). ExPASy: the proteomics server for in-depth protein knowledge and analysis. Nucleic Acid Res.

[70] Katoh K, Standley DM (2013). MAFFT multiple sequence alignment software version 7: improvements in performance and usability. Mol Biol Evol.

[71] Guindon S, Dufayard JF, Lefort V, Anisimova M, Hordijk W, Gascuel O (2010). New algorithms and methods to estimate maximum-likelihood phylogenies: assessing the performance of PhyML 3.0. Syst Biol.

[72] Le SQ, Gascuel O (2008). LG: an improved, general amino-acid replacement matrix. Mol Biol Evol.

