# Supplementary material for: Phylogenetic analysis reveals dynamic evolution of the poly(A)-binding protein gene family in plants
Source: BMC Evol Biol. 2014 Nov 25;14:238. doi: 10.1186/s12862-014-0238-4 (PMC4252990; doi:10.1186/s12862-014-0238-4)
Supplement: Additional file 1: — Phylogenetic analysis of PABP proteins in plants. A phylogenetic tree of PABP proteins in plants was constructed using the maximum-likelihood method. Numbers on each branch denote percentages of bootstrap support. PABP sequences from yeast and humans were used to root the tree. [file 12862_2014_238_MOESM1_ESM.pdf]

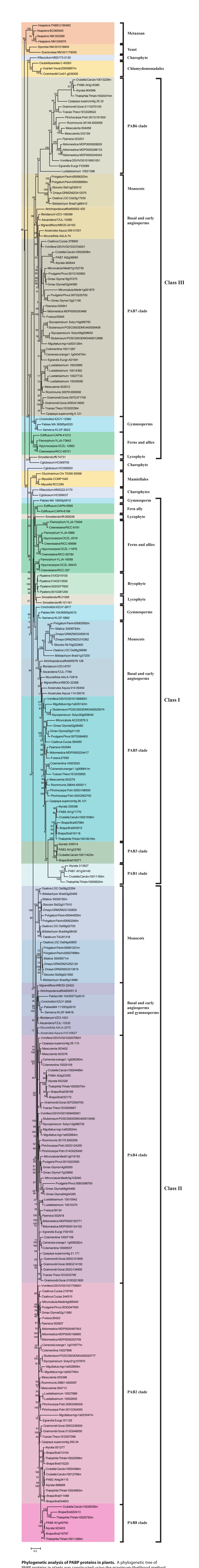

Phylogenetic analysis of PABP proteins in plants. A phylogenetic tree of PABP proteins in plants was constructed using the maximum-likelihood method. Numbers on each branch denote percentages of bootstrap support. PABP sequences from yeast and humans were used to root the tree.

Additional file 1
