# Supplementary material for: Phylogenetic analysis reveals dynamic evolution of the poly(A)-binding protein gene family in plants
Source: BMC Evol Biol. 2014 Nov 25;14:238. doi: 10.1186/s12862-014-0238-4 (PMC4252990; doi:10.1186/s12862-014-0238-4)
Supplement: Additional file 2: — Phylogenetic relationships among the plant species used in this study. Plant groups and their relationships are shown to the left with the corresponding species included in the analysis listed to the right. [file 12862_2014_238_MOESM2_ESM.pdf]

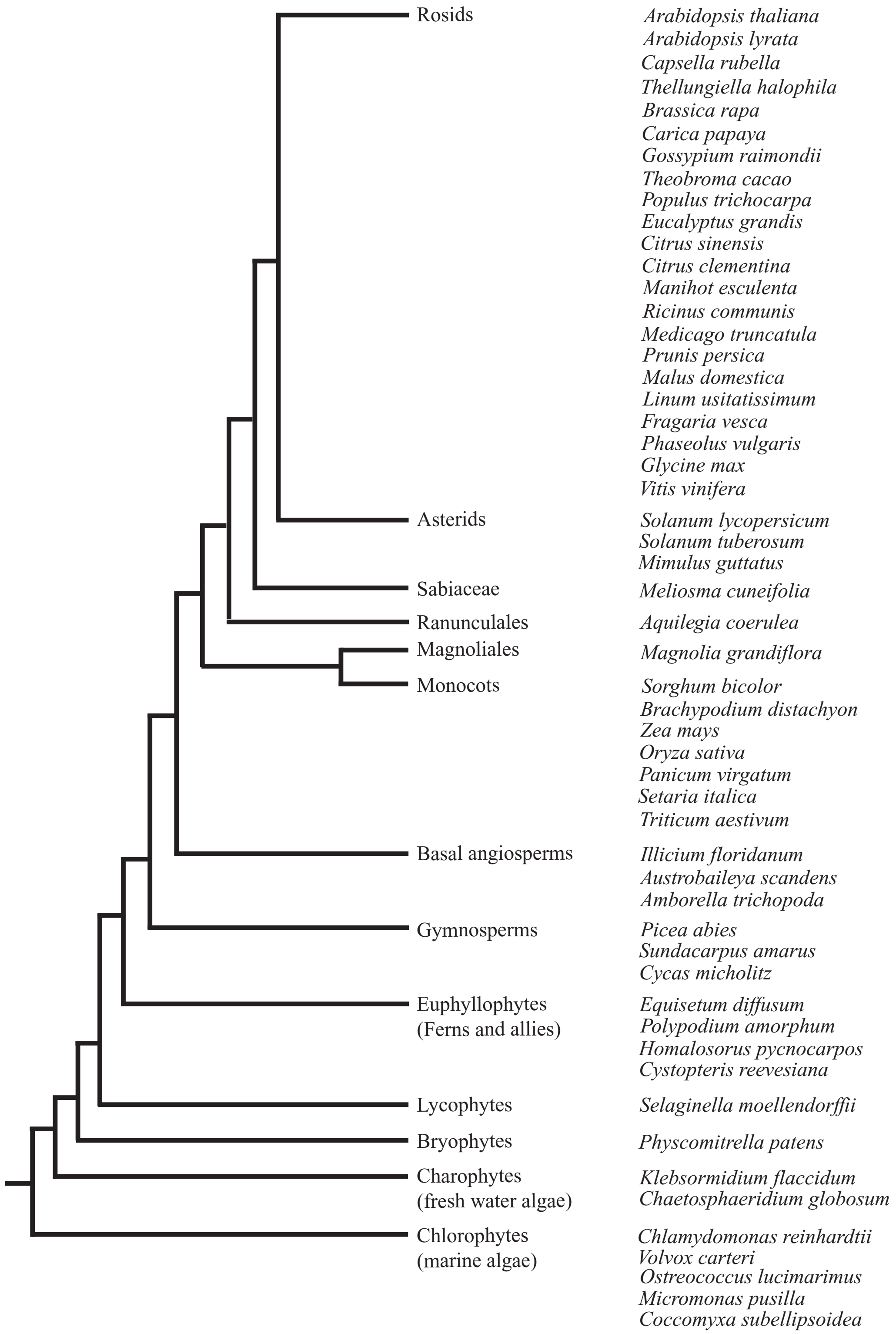

Phylogenetic relationships among the plant species used in this study. Plant groups and their relationships are shown to the left with the corresponding species included in the analysis listed to the right.
